# Feeding Agroindustrial Byproducts to Light Lambs: Influence on Growth Performance, Diet Digestibility, Nitrogen Balance, Ruminal Fermentation, and Plasma Metabolites

**DOI:** 10.3390/ani10040600

**Published:** 2020-04-01

**Authors:** Trinidad de Evan, Almudena Cabezas, Jesús de la Fuente, María Dolores Carro

**Affiliations:** 1 Departamento de Producción Agraria, ETSIAAB, Universidad Politécnica de Madrid, Ciudad Universitaria, 28040 Madrid, Spain; t.deevan@alumnos.upm.es; 2 Departamento de Producción Animal, Facultad de Veterinaria, Universidad Complutense de Madrid, 28040 Madrid, Spain; almucabe@ucm.es (A.C.); jefuente@vet.ucm.es (J.d.l.F.)

**Keywords:** light lambs, corn DDGS, citrus pulp, exhausted olive cake, growth performance, amino acids

## Abstract

**Simple Summary:**

Feeding agroindustrial byproducts to ruminants can have multiple benefits, such as lowering feeding costs, reducing competition with human food, decreasing environmental impact associated with byproducts disposal, and improving the quality of animal products. In order to use these byproducts in practical feeding, their effects on animal performance and health should be assessed. In this study, we evaluated the effect of replacing 44% of conventional feeds in a high-cereal concentrate for light lambs with three byproducts: Corn distiller’s dried grains with solubles (18%), dried citrus pulp (18%), and exhausted olive cake (8%), all of which are highly produced in the Mediterranean area. We observed that the inclusion of these byproducts did not affect feed intake, growth performance, ruminal fermentation (with exception of NH_3_-N concentrations), or plasma metabolites in growing lambs. Compared with the high-cereal concentrate, feeding the concentrate including the byproducts resulted in a reduction of potentially human-edible ingredients from 64.4% to 38.7%. In conclusion, 44% of cereal grains and protein feeds in the concentrate for light lambs can be replaced with a mixture of corn distiller’s dried grains with solubles, dried citrus pulp, and exhausted olive cake without negatively affecting growing performance and animal health.

**Abstract:**

The objective of this study was to evaluate the effect of replacing cereals and protein concentrates in a high-cereal concentrate (control) for light lambs with corn distiller’s dried grains with solubles (DDGS; 18%), dried citrus pulp (DCP; 18%), and exhausted olive cake (EOC; 8%) in a byproduct (BYP) concentrate on growth performance, digestibility, ruminal fermentation, and plasma metabolites. Two homogeneous groups of Lacaune lambs (13.8 kg ± 0.25 kg) were fed one of each concentrates and barley straw *ad libitum* until reaching about 26 kg body weight. There were no differences between groups on feed intake, average daily gain, or feed conversion ratio, but the control diet had greater (*p* < 0.001) dry matter digestibility. Diet had no effect on post-mortem ruminal pH and total volatile fatty acid concentrations and profile, but NH_3_-N concentrations were lower (*p* = 0.003) for the BYP-fed group compared with the control one. However, plasma concentrations of amino acids, total proteins, urea, and hepatic enzymes were not affected by the diet. In conclusion, 44% of feed ingredients in the concentrate for light lambs can be replaced with a mixture of corn DDGS, DCP, and EOC without negatively affecting growing performance and animal health.

## 1. Introduction

Agroindustrial byproducts have been traditionally used for small ruminant feeding in low-input systems, but their use is continuously increasing worldwide. Although either the shortage or the high cost of conventional feeds has been the main reason for feeding byproducts to livestock, other reasons are equally or even more important to support their current use. Some byproducts are highly contaminant, and their use in animal feeding can help to reduce the environmental problems caused by their accumulation and to lower the carbon footprint of animal products when locally produced byproducts are used [1]. Most byproducts are not potentially edible by humans, and they therefore do not compete directly with human food [2]. Additionally, some byproducts contain bioactive compounds that can improve animal health and the quality of animal products [3,4] while contributing to farm sustainability.

Three byproducts originated in the olive oil, citrus, and ethanol industries, all highly produced in the Mediterranean area, were used in this study. Olive oil production has tripled in the last 60 years with an estimated production of 3.1 × 10^6^ t for the 2019/20 crop year [5] and is concentrated in the Mediterranean area, with Spain being the first world producer and exporter [6]. The two-phase system used to obtain olive oil generates a high-moisture byproduct named “alperujo,” which can be partially destoned, dried, and subjected to a chemical extraction to obtain pomace olive oil in a process that generates “exhausted olive cake” (EOC) as a waste [7]. Citrus fruits are produced worldwide, and about 70% of total production is grown in the Northern Hemisphere (mainly in the USA and countries in the Mediterranean area), although Brazil is the largest producer [8]. The main byproduct of citrus industry is citrus pulp, a high-moisture product that can be dried (DCP) and used in ruminant feeding as energy source [9]. Both EOC and DCP are rich in polyphenols [7,9,10], and they may therefore modify ruminal fermentation and improve animal health. Dried distillers grains with solubles (DDGS) are byproducts of the ethanol industry. Whereas there is a lot of information available on the effects of DDGS in dairy cows feeding, their influence on lamb growth has been much less studied. The objective of this study was therefore to assess the effects of replacing conventional feed ingredients in a concentrate with a mixture of EOC, DCP, and DDGS on feed intake, growth performance, nutrient digestibility, nitrogen (N) balance, ruminal fermentation, and plasma metabolites in light lambs.

## 2. Materials and Methods

The lambs used in this trial were cared for and handled in accordance with the Spanish guidelines for experimental animal protection, and experimental procedures were approved by the General Direction of Livestock and Agriculture of the Community of Madrid (Approval number PROEX 035/17).

### 2.1. Animals and Experimental Diets

Twenty four Lacaune male lambs, with an initial body weight (BW) of 13.8 ± 0.25 kg, were homogeneously distributed in two groups according to their BW. Lambs were penned individually in 1-m x 1-m pens with slatted floor, which were placed 1 m above the floor and equipped with two feeders and an automatic drinker. Each group of lambs was randomly assigned to one of the two dietary treatments: A high-cereal concentrate (control) and a byproduct containing concentrate (BYP), in which 44% of conventional feed ingredients (corn, barley, soybean meal, palm meal, and wheat bran) were replaced with corn DDGS, DCP, and EOC (18%, 18%, and 8% of concentrate, respectively, as-fed basis). The ingredients and chemical composition of both concentrates is shown in Table 1. Corn DDGS and DCP were commercially available and EOC was obtained from an extraction plant located in the south of Spain (Puente Genil, Córdoba). Concentrate ingredients were ground and pelleted (4-mm size). The concentrates were formulated to meet the nutritive requirements of light lambs [11]. Both concentrates were formulated to have similar crude protein (CP) and neutral-detergent fiber (NDF) content. Lambs were fed *ad libitum* concentrate and barley straw and had free access to fresh water over the trial. The barley straw contained (as-fed basis) 92.3%, 7.25%, 2.90%, 1.60%, 71.9%, and 38.0% of dry matter (DM), ashes, CP, ether extract (EE), NDF, and acid-detergent fiber (ADF), respectively. According to INRA [11], 1 kg of barley straw has 0.30 forage units for meat production (UFV).

### 2.2. Experimental Procedure and Measurements

The experiment lasted for six weeks and included seven days for diet adaptation. Concentrate and straw intakes were measured twice per week, whereas BW of all lambs was determined weekly. Samples of offered concentrate and straw were taken weekly for analysis of chemical composition. On days 0, 21, and the slaughter day, blood samples were taken from each lamb by jugular venipuncture into tubes containing EDTA immediately before feeding. Samples were centrifuged (5000× *g*, 10 min, 4 °C), and the plasma was immediately frozen (−20 °C) until determination of concentrations of amino acids, albumin, globulins, total proteins, urea, and cholesterol, the activities of the enzymes lactate dehydrogenase (LDH), alkaline phosphatase (ALP), creatine phosphokinase (CPK), glutamic oxaloacetic transaminase (GOT), glutamic pyruvic transaminase (GPT), and gamma-glutamyl transpeptidase (GGT).

In the fourth week of the trial, the digestibility of the diets and the nitrogen balance were measured in nine lambs per treatment. Trays were placed under the slated floor of each pen for feces and urine collection. The trays had holes for urine collection, which was collected in a bucket containing a solution of H_2_SO_4_ (10%, vol/vol) to keep the pH below 3.0 [12]. The feces and urine voided by each lamb in 24 h were quantitatively collected for six days and aliquots (10%) were sampled daily for digestibility and N balance determination, respectively. Daily samples were pooled to form a composite sample for feces and urine for each lamb, which was frozen until analysis.

In the last week of the trial, lambs were slaughtered at a commercial slaughterhouse located 20 km away from the experimental farm on two different days. The six lambs of each treatment with the greatest body weight were slaughtered the first day, and the rest of lambs the second day. Lambs had free access to feed and water until about 2 h before slaughter, and were slaughtered according to commercial practices involving head electrical stunning and severing the carotid arteries and jugular veins. After slaughter and dressing, the full gastrointestinal tract was removed and samples from rumen contents were immediately taken. The ruminal content was homogenized, a sample of about 300 g was filtered through four layers of gauze, and the pH of the fluid was immediately measured using a Crisson Basic 20 pH-meter (Crisson Instruments, Barcelona, Spain). Then, 2 mL of fluid were mixed with 2 mL of 0.5 N HCl and samples were frozen (−20°C) until analyses of volatile fatty acids (VFA) and NH_3_-N concentrations. In addition, the color of the rumen epithelium was evaluated as described by Haro [13]. Briefly, a sample (10 × 10 cm) of the rumen wall was collected from the ventral area of the rumen after empting the rumen content. Samples were washed with saline solution, displayed on a white surface under an intense and homogeneous light, and the color was evaluated using a scale from 1 to 5. The lightest epithelium received a score of 1 and the darkest one received a score of 5, whereas the rest of the samples were assigned scores according to their color intensity. The evaluation was performed by four trained persons (blind to treatment allocation), and the average score was used for statistical analysis. 

### 2.3. Chemical Analyses

The procedures of the Association of Official Analytical Chemists [14] were used to analyze the DM (method 934.01), ash (method 942.05), and EE (method 920.39) content in samples of diet ingredients and feces. The N content of diet ingredients, feces, and urine was determined according to the Dumas method using a TruSpec CN equipment (Leco Corp. St. Joseph, MI, USA). Analysis of NDF in feed ingredients and feces was carried out according to Van Soest et al. [15] and that of ADF and lignin according to Robertson and Van Soest [16]. All fiber analyses were carried out using an Ankom 220 Fiber Analyzer unit (Ankom Technology Corp., Macedon, NY, USA), and results were expressed exclusive of residual ash. Concentrations of VFA in ruminal fluid were analyzed by gas chromatography using a Shimadzu GC 2010 chromatography (Shimazdu Europa GmbH, Duisburg, Germany) provided with a TR-FFAP column (30 m × 0.53 mm × 1 µm; Supelco, Madrid, Spain) according to the procedure described by García-Martinez [17], whereas NH_3_-N concentrations were determined by the phenol-hypochlorite method of Wheatherburn [18].

Plasma concentrations of amino acids were determined by high-performance liquid chromatography (HPLC) as described by Frank and Powers [19], whereas those of albumin, globulins, total proteins, urea, cholesterol, and the enzymes LDH, ALP, CPK, GOT, GPT, and GCT were determined using an automatic biochemistry analyzer (Hitachi 7020; Hitachi High Technologies, Inc., Ibaraki, Japan).

### 2.4. Statistical Analyses

Data on feed intake, growth performance, ruminal fermentation, meat composition, digestibility, and N balance were analyzed with one-way analysis of variance using the GLM PROC of the SAS [20]. Data on plasma concentrations of amino acids and metabolites were analyzed as a mixed model with repeated measures over time using the PROC MIXED of SAS [20]. The model included the diet, time, and their interaction as fixed effects, and the lamb as random effects. The level of statistical significance was set at *p* < 0.05, and *p* values between 0.05 and 0.10 were considered trends.

## 3. Results and Discussion

The proportion of each byproduct in the diet was selected from the results of in vitro studies by our group [21] and published in vivo studies [9,10]. The DCP contained (as-fed basis) 6.25%, 4.86%, 17.9%, and 3.28% of ashes, CP, NDF, and EE, respectively, whereas these values were 5.32%, 26.4%, 37.1%, and 9.81% for corn DDGS, and 14.0%, 7.48%, 46.4%, and 2.91% for EOC. The chemical composition of the used byproducts was within the range of values reported in the literature [7,11]. The two concentrates were formulated to have similar CP and NDF contents, but the BYP concentrate resulted in slightly greater CP amount than expected. The proportion of potentially human-edible ingredients in each concentrate was calculated as proposed by Wilkinson [22], and it was decreased from 64.4% in the control concentrate to 38.7% in the BYP concentrate, showing that feeding BYP to lambs resulted in lower competition with human nutrition.

### 3.1. Feed Intake, Growht Performance, Diet Digestibility, and Nitrogen Balance

As shown in Table 2, there were no differences between the two experimental groups in either concentrate or straw intake, indicating that BYP palatability was not negatively affected by the inclusion of byproducts. Barley straw intake was low in both groups, averaging 4.64% and 5.29% of the total DM intake for the control and BYP-fed lambs, respectively, which is in agreement with the low straw intake observed in previous studies by our group in light lambs under similar feeding conditions [12,13]. Both concentrates had similar NDF content (Table 1), but the fiber of DDGS and DCP is rapidly degraded in the rumen [23] and it might therefore be less effective at stimulating rumination. However, the lambs fed the BYP concentrate did not augment their intake of straw to increase their intake of physically effective fiber.

There were no differences between groups in final BW, average daily gain (ADG), and feed conversion rate. In agreement with these results, feeding the BYP concentrate did not affect carcass weights (*p* ≥ 0.704) or carcass yield compared with control concentrate. Values for these parameters were in the range previously reported by others for light lambs fed high-cereal concentrates and slaughtered at about 25–26 kg BW [12,13,24,25]. The lack of differences between the groups in growth performance is consistent with their similar energy intake, which averaged 0.840 and 0.834 daily forage units for meat production (UFV) for the control and BYP groups, respectively.

The digestibility of both DM and organic matter (OM) was greater (*p* < 0.001) for control than for BYP concentrate (Table 3), but there were no differences (*p* ≥ 0.120) between concentrates in the digestibility of CP, NDF, and ADF. The lower DM and OM digestibility of the BYP concentrate may have been partly due to its lower content in non-structural carbohydrates (35.2% vs. 45.7% for BYP and control concentrates, respectively), which is a highly digestible fraction, and its greater lignin content (1.79% vs. 2.80%). Values of diet digestibility were in the range reported by others for lambs fed high-cereal diets [12,13,26,27] and diets including olive cake [28] or dehydrated citrus pulp [29].

The greater (*p* = 0.024) N intake of the BYP-fed lambs was due to the greater CP content of BYP compared with the control concentrate, as there was no differences between groups in concentrate intake (Table 3). The daily fecal N excretion of BYP group was also greater (*p* = 0.012) than that for control group, but differences disappeared when fecal N excretion was expressed as proportion of the ingested N, which is consistent with the lack of differences in CP digestibility. There were no differences between groups in the amount of urinary and retained N, either expressed as g/d or as a proportion of the ingested N. Values of N retention were in the range of those previously reported for young growing lambs fed diets with similar CP levels [12,30].

### 3.2. Ruminal Fermentation and Plasma Metabolites

There was no effect (*p* ≥ 0.247) of the type of concentrate on *post-mortem* ruminal pH, total VFA concentrations, molar proportions of individual VFA, or acetate/propionate ratio (Table 4). The pH values and acetate/propionate ratios were similar to those reported for lambs fed high-cereal concentrates and straw [13,26,31,32]. The lower (*p* = 0.003) NH_3_-N concentrations in the rumen of BYP-fed lambs compared with control-fed lambs might indicate lower CP degradability in the BYP-concentrate. In fact, DDGS protein has lower ruminal degradability than other concentrate feeds, such as soybean and palm meal [23,33], which were replaced in the BYP-concentrate. In addition, 50.8% of the CP in the EOC was bound to the ADF, indicating low N availability [23]. Concentrations of NH_3_-N in the rumen of control-fed lambs were adequate for microbial growth [34], but those in BYP-fed lambs might have been limiting. However, the lack of differences in any of the ruminal parameters measured precludes this hypothesis. 

The reasons for the trend (*p* = 0.092) to darker color of the ruminal epithelium in the lambs fed the BYP-concentrate are unclear. A darker color of ruminal epithelium is usually associated to keratinized tissue [35], but the lack of differences between the two groups of lambs in both growth performance and most ruminal parameters indicate that ruminal absorption was not negatively affected in the BYP-fed lambs.

There were no effects (*p* ≥ 0.109) of the diet on plasma concentrations of any amino acid, and no diet x sampling day interactions (*p* ≥ 0.151) were detected (Table 5). These results are consistent with the lack of differences between groups in growth rates and CP digestibility, and indicate no amino acid limitation in the BYP group compared with the control one. Changes in plasma amino acids due to dietary modifications were the consequence of modifications in the flow of total amino acids into the duodenum, including amino acids from both dietary and microbial origin. The lack of differences between groups in plasma amino acids concentrations supports the hypothesis that microbial protein synthesis was not limited in BYP-fed lambs, despite the low ruminal NH_3_-N concentrations. In agreement with previous studies [36], the concentrations of most amino acids increased or tended (*p* ≤ 0.065) to increase as lambs grew older, with the exception of glutamic acid, histidine, glycine, arginine, alanine, and cysteine, which did not change over the trial. The increased amino acids concentrations at the end of the trial are in accordance with the greater growth rate of the lambs at this time, as amino acids are used for protein synthesis and tissue formation [37].

No diet x time interactions (*p* ≥ 0.223) were detected in any of the metabolites measured in the blood of lambs (Table 6). The lack of differences between diets in plasma concentrations of urea (*p* = 0.585) is in accordance with the similar concentrations of amino acids observed in both groups of lambs. Plasma urea concentrations reflect the amount of urea produced in the liver from amino acid catabolism [38], although they were also highly affected by the amount of NH_3_-N absorbed from the rumen. The similar (*p* ≥ 0.276) levels of albumin, globulins, and total proteins in the plasma of both groups agree well with the lack of differences in plasma concentrations of amino acids and urea. Plasma concentrations of these metabolites, excluding globulins, increased with time. The normal [39,40,41] and similar levels of cholesterol and enzymes activities (LDH, ALP, CPK, GOT, GPT, and CGT) observed in both groups over the trial indicate that feeding the BYP diet did not negatively affect liver function.

Finally, it is worth mentioning that under the conditions of our study, the cost of the control and BYP concentrates were 0.270 €/kg and 0.262 €/kg, respectively, resulting in a calculated cost of concentrate per kg of gain was 0.787 € and 0.777 € per control and BYP concentrate, respectively. However, these results may vary, as feed prices are highly volatile.

## 4. Conclusions

Our results indicate that a mixture of corn DDGS, DCP and EOC can replace 44% of cereal grains and protein feeds in the concentrate for light lambs without negatively affecting the feed intake, growth performance, or health of lambs. The use of these byproducts reduced the potentially human-edible ingredients in the concentrate from 64.4% to 38.7% and slightly decreased the feeding costs. In situations of high prices of cereals and protein feeds, the use of byproducts by lamb farmers can be more economically profitable than in the present study.

## Figures and Tables

**Table 1:** Ingredient and chemical composition of experimental concentrates.

Item	Control	BYP
Ingredients (% as fed)		
Corn	33.0	26.8
Barley	20.0	-
Wheat	10.0	10.0
Soybean meal 47%	12.2	10.2
Palm meal	8.8	-
Colza meal	2.5	2.5
Wheat bran	10.0	3.0
Dry citrus pulp	-	18.0
Corn DDGS	-	18.0
Olive cake	-	8.0
Others ^1^	3.5	3.5
Chemical composition (%, as-fed basis)		
Dry matter (DM)	89.7	88.6
Ashes	4.82	5.97
Crude protein (CP)	16.2	17.5
Ether extract (EE)	3.75	6.44
Neutral detergent fiber (NDF)	19.2	19.5
Acid detergent fiber (ADF)	7.47	9.31
Acid detergent lignin	1.79	2.80
Non-structural carbohydrates (NSC) ^2^	45.7	35.2
Forage units for meat production (UFV) ^3^	1.00	0.96

^1^ For both concentrates: 1.2% Calcium soap; 1.0% calcium carbonate; 0.8% sodium bicarbonate; 0.3% NaCl, and 0.2% vitamin-mineral premix; ^2^ Calculated as DM − (ashes + CP + EE + NDF); ^3^ Calculated according to INRA [11].

**Table 2:** Initial and final body weight, feed intake, average daily gain, feed conversion rate, carcass weights, and carcass yield of light lambs fed either a high-cereal concentrate (control) or a concentrate including byproducts (BYP) ^1^.

Item	Control (n = 12)	BYP (n = 12)	SEM ^2^	*p*-Value
Initial body weight (kg)	13.6	13.9	0.95	0.595
Feed intake (g/d)				
Concentrate	828	854	77.9	0.568
Straw	40.3	47.7	13.40	0.341
Total	869	902	77.9	0.463
Final body weight (kg)	26.2	26.4	1.25	0.744
Average daily gain (g/d)	284	288	32.9	0.844
Feed conversion rate (g concentrate/g)	2.92	2.97	0.320	0.747
Carcass traits				
Hot carcass weight (kg)	14.4	14.2	0.76	0.704
Cold carcass weight (kg)	13.6	13.6	0.90	0.987
Cold carcass yield (%)	51.9	51.5	2.15	0.765

^1^ BYP concentrate contained 18% corn DDGS, 18% dried citrus pulp and 8% exhausted olive cake (as-fed basis); ^2^ Standard error of the mean.

**Table 3:** Diet digestibility and nitrogen balance in light lambs fed either a high-cereal concentrate (control) or a concentrate including byproducts (BYP) ^1^.

Item	Control (n = 9)	BYP (n = 9)	SEM ^2^	*p*-Value
Digestibility (%)				
Dry matter	79.1	74.4	0.74	< 0.001
Organic matter	81.4	76.7	0.69	< 0.001
Crude protein	75.2	73.1	0.97	0.141
Neutral detergent fiber	53.3	54.8	1.80	0.570
Acid detergent fiber	47.6	52.0	1.91	0.120
Nitrogen (N) balance				
N intake (g/d)	21.8	24.1	0.66	0.024
Fecal N				
g/d	5.40	6.49	0.273	0.012
% of ingested N	24.8	26.9	0.969	0.141
Urinary N				
g/d	4.73	5.17	0.598	0.616
% of ingested N	21.4	21.4	2.28	0.988
Retained N				
g/d	11.7	12.5	0.55	0.312
% of ingested N	53.8	51.7	2.21	0.519

^1^ BYP concentrate contained 18% corn DDGS, 18% dried citrus pulp and 8% exhausted olive cake (as-fed basis); ^2^ Standard error of the mean.

**Table 4:** Ruminal parameters measured post-mortem and rumen wall color in light lambs fed either a high-cereal concentrate (control) or a concentrate including byproducts (BYP) ^1^.

Item	Control (n = 12)	BYP (n = 12)	SEM ^2^	*p*-Value
pH	5.40	5.62	0.126	0.263
NH_3_-N (mg/l)	69.2	45.7	2.17	0.003
Total volatile fatty acids (VFA; mM)	140	130	15.1	0.643
Molar proportions (mol/100 mol)				
Acetate	52.8	52.8	1.71	0.987
Propionate	33.2	33.6	2.17	0.900
Butyrate	9.26	8.18	0.939	0.424
Isobutyrate	0.58	0.45	0.090	0.332
Isovalerate	0.67	0.54	0.104	0.388
Valerate	3.01	3.80	0.297	0.073
Caproate	0.56	0.68	0.131	0.516
Acetate/propionate ratio (mol/mol)	1.77	1.64	0.175	0.593
Rumen wall colour ^3^	2.19	3.19	0.402	0.092

^1^ BYP concentrate contained 18% corn DDS, 18% dried citrus pulp and 8% exhausted olive cake (as-fed basis); ^2^ Standard error of the mean; ^3^ Scored from 1 (pale) to 5 (dark).

**Table 5:** Plasma concentrations of amino acids in light lambs fed either control concentrate (CON; n = 12) or a concentrate including byproducts (BYP; n = 12) at the beginning (day 0) and end (day 42) of the trial ^1^.

Item	Diet	Day		*p*-Value			
		0	42	SEM ^2^	Diet	Day	Diet × Day
Amino acid (µmol/L)							
Aspartic acid	CON	34.7	39.0	1.23	0.909	<0.001	0.156
	BYP	33.1	41.4				
Glutamic acid	CON	177	205	22.8	0.608	0.162	0.767
	BYP	186	227				
Asparagine	CON	41.1	46.6	4.57	0.742	0.065	0.439
	BYP	35.7	48.8				
Serine	CON	57.4	75.6	6.97	0.547	0.062	0.597
	BYP	65.7	76.2				
Glutamine	CON	57.5	69.6	6.86	0.902	0.053	0.721
	BYP	55.8	73.2				
Histidine	CON	35.4	38.7	2.36	0.238	0.132	0.841
	BYP	31.5	35.8				
Glycine	CON	274	295	32.5	0.312	0.871	0.433
	BYP	336	303				
Threonine	CON	77.8	154	14.08	0.826	<0.001	0.631
	BYP	88.5	150				
Arginine	CON	112	139	13.5	0.368	0.343	0.340
	BYP	112	112				
Alanine	CON	63.3	67.0	6.51	0.760	0.654	0.926
	BYP	66.7	69.2				
Tyrosine	CON	58.5	72.2	6.62	0.971	0.010	0.403
	BYP	53.4	78.8				
Cysteine	CON	203	199	18.8	0.824	0.786	0.914
	BYP	213	205				
Valine	CON	103	155	16.5	0.280	<0.001	0.267
	BYP	102	194				
Methionine	CON	32.5	41.8	2.42	0.859	<0.001	0.223
	BYP	29.7	45.5				
Tryptophan	CON	28.0	35.2	1.67	0.294	<0.001	0.387
	BYP	25.4	31.8				
Phenylalanine	CON	47.0	53.8	4.12	0.741	0.023	0.385
	BYP	45.2	59.6				
Isoleucine	CON	57.5	70.4	7.67	0.483	0.021	0.375
	BYP	56.0	83.4				
Leucine	CON	63.8	99.1	11.24	0.109	<0.001	0.151
	BYP	66.7	137				
Lysine	CON	93.9	118	10.21	0.953	0.005	0.406
	BYP	83.9	127				
Essential AA	CON	539	766	63.7	0.531	<0.001	0.422
	BYP	528	865				
Total AA	CON	1752	2134	189.3	0.654	0.064	0.934
	BYP	1833	2249				

^1^ BYP concentrate contained 18% corn DDGS, 18% dried citrus pulp and 8% exhausted olive cake (as-fed basis); ^2^ Standard error of the mean.

**Table 6:** Plasma concentrations of metabolites in light lambs fed either a high-cereal concentrate (CON; n = 12) or a concentrate including byproducts (BYP; n = 12) over the trial ^1^.

Item ^2^	Diet	Day					*p*-Value		
		0	21	42	SEM_d_^3^	SEM_sd_^3^	Diet	Day	Diet × Day
Urea (mg/100 mL)	CON	22.9 ^ab^	20.3 ^a^	25.4 ^b^	0.71	0.86	0.585	0.004	0.889
	BYP	23.1 ^ab^	22.0 ^a^	25.8 ^b^					
Albumine (mg/100 mL)	CON	2.12 ^a^	2.30 ^a^	2.53 ^b^	0.040	0.049	0.276	<0.001	0.571
	BYP	2.28 ^a^	2.32 ^a^	2.59 ^b^					
Globulins (g/100 mL)	CON	3.18	3.32	3.55	0.097	0.118	0.556	0.316	0.742
	BYP	3.16	3.27	3.30					
Total proteins (g/100 mL)	CON	5.30 ^a^	5.62 ^ab^	6.00 ^b^	0.095	0.117	0.692	0.003	0.483
	BYP	5.44 ^ab^	5.38 ^a^	5.88 ^b^					
Cholesterol (mg/100 mL)	CON	54.3 ^b^	38.8 ^a^	40.1 ^a^	1.94	2.38	0.876	<0.001	0.952
	BYP	53.8 ^b^	37.3 ^a^	40.6 ^a^					
LDH (Units/L)	CON	502 ^a^	670 ^b^	653 ^b^	12.8	15.7	0.638	<0.001	0.538
	BYP	495 ^a^	706 ^b^	673 ^b^					
ALP (Units/L)	CON	2.15 ^a^	1.45 ^a^	5.08 ^b^	0.293	0.359	0.592	<0.001	0.922
	BYP	2.18 ^a^	1.70 ^a^	5.30 ^b^					
CPK (Units/L)	CON	102 ^a^	201 ^b^	123 ^a^	9.9	12.1	0.780	<0.001	0.954
	BYP	96.6 ^a^	203 ^b^	114 ^a^					
GOT (Units/L)	CON	45.2 ^a^	70.8 ^b^	72.5 ^b^	2.18	2.675	0.522	<0.001	0.889
	BYP	45.6 ^a^	72.5 ^b^	76.6 ^b^					
GPT (Units/L)	CON	5.62 ^a^	13.1 ^c^	9.54 ^b^	0.436	0.534	0.755	<0.001	0.738
	BYP	6.09 ^a^	12.6 ^c^	8.92 ^b^					
GGT (Units/L)	CON	101	98.1	106	3.43	4.20	0.114	0.215	0.223
	BYP	102	122	117					

^a,b,c^ Within a row, means with different superscript differ (*p* < 0.05); ^1^ BYP concentrate contained 18% corn DDGS, 18% dried citrus pulp and 8% exhausted olive cake (as-fed basis); ^2^ LDH: Lactate dehydrogenase, ALP: Alkaline phosphatase, CPK: creatine phosphokinase, GOT: Glutamic oxaloacetic transaminase, GPT: Glutamic pyruvic transaminase, GCT: Gamma-glutamyl transpeptidase; ^3^ SEM_d_ and SEM_sd_: Standard error of the mean for diet and sampling day effects, respectively.

## References

[1] Gerber P.J., Uwizeye A., Schulte R.P.O., Opio C.I., de Boer I.J.M. (2014). Nutrient use efficiency: A valuable approach to benchmark the sustainability of nutrient use in global livestock production?. Curr. Opin. Environ. Sustain..

[2] Bakshi M.P.S., Wadhwa M., Makkar H. (2016). Waste to worth: Vegetable wastes as animal feed. Cab. Rev..

[3] Vasta V., Nudda A., Cannas A., Lanza M., Priolo A. (2008). Alternative feed resources and their effects on the quality of meat and milk from small ruminants. Anim. Feed Sci. Technol..

[4] Correddu F., Lunesu M.F., Buffa G., Atzori A.S., Nudda A., Battacone G., Pulina G. (2020). Can Agro-Industrial By-Products Rich in Polyphenols be Advantageously Used in the Feeding and Nutrition of Dairy Small Ruminants?. Animals.

[5] International Olive Oil Organization Olive Oil Estimates 2019/20 Crop Year. https://www.internationaloliveoil.org/olive-oil-estimates-2019-20-crop-year/.

[6] MAPA 2020 Ministerio de Agricultura, Pesca y Alimentación. http://www.mapa.gob.es/.

[7] Marcos C.N., García-Rebollar P., de Blas C., Carro M.D. (2019). Variability in the Chemical Composition and In Vitro Ruminal Fermentation of Olive Cake By-Products. Animals.

[8] UNCTD (2020). United Nations Conference on Trade and Development. https://unctad.org/SearchCenter/Pages/results.aspx?k=citrus%20production.

[9] Bampidis V.A., Robinson P.H. (2006). Citrus by-products as ruminant feeds: A review. Anim. Feed Sci. Technol..

[10] Molina-Alcaide E., Yáñez-Ruíz D.R. (2008). Potential use of olive by-products in ruminant feeding: A review. Anim. Feed Sci. Technol..

[11] Sauvant D., Delaby L., Noziere P., Noziere P., Sauvant D., Delaby L. (2017). INRA Feeding System for Ruminants.

[12] Carro M.D., Ranilla M.J., Giráldez F.J., Mantecón A.R. (2006). Effects of malate supplementation on feed intake, digestibility, microbial protein synthesis and plasma metabolites in lambs fed a high-concentrate diet. J. Anim. Sci..

[13] Haro A.N., González J., de Evan T., de la Fuente J., Carro M.D. (2019). Effects of feeding rumen-protected sunflower seed and meal protein on feed intake, diet digestibility, ruminal and cecal fermentation, and growth performance of lambs. Animals.

[14] Association of Official Analytical Chemists (AOAC) (2005). Official Methods of Analysis.

[15] Van Soest P.J., Robertson J.B., Lewis B.A. (1991). Methods for dietary fiber, neutral detergent fiber and nonstarch polysaccharides in relation to animal nutrition. J. Dairy Sci..

[16] Robertson J.B., Van Soest P.J., James W.P.T., Theander O. (1981). The detergent system of analysis and its application to human foods. The Analysis of Dietary Fiber in Food.

[17] García-Martínez R., Ranilla M.J., Tejido M.L., Carro M.D. (2005). Effects of disodium fumarate on in vitro rumen microbial growth, methane production and fermentation of diets differing in their forage concentrate ratio. Br. J. Nutr..

[18] Weatherburn M.W. (1967). Phenol-hypochlorite reaction for determination of ammonia. Anal. Chem..

[19] Frank M.P., Powers R.W. (2007). Simple and rapid quantitative high-performance liquid chromatographic analysis of plasma amino acids. J. Chromatogr. B. Analyt. Technol. Biomed. Life Sci..

[20] SAS Institute (2017). SAS/STAT® Users Guide, Version 9.3.

[21] Jiménez C. (2018). Use of Agroindustrial by-Products (Olive Cake, Tomato Pulp and Wine Lees) in Diets for Fattening Lambs: In Vitro Evaluation. Master’s Thesis.

[22] Wilkinson J.M. (2011). Re-defining efficiency of feed use by livestock. Animal.

[23] NRC (National Research Council) (2001). Nutrient Requirements of Dairy Cattle.

[24] Manso T., Mantecón A.R., Giráldez F.J., Lavín P., Castro T. (1998). Animal performance and chemical body composition of lambs fed diets with different protein supplements. Small Rum. Res..

[25] Carrasco S., Ripoll G., Sanz A., Álvarez-Rodríguez J., Panea B., Revilla R., Joy M. (2009). Effect of feeding system on growth and carcass characteristics of Churra Tensina light lambs. Livest. Sci..

[26] Blanco C., Giráldez F.J., Prieto N., Morán L., Andrés S., Benavides J., Tejido M.L., Bodas R. (2014). Effects of dietary inclusion of sunflower soap stocks on nutrient digestibility, growth performance, and ruminal and blood metabolites of light fattening lambs. J. Anim. Sci..

[27] Blanco C., Bodas R., Prieto N., Andrés S., López S., Giráldez F.J. (2014). Concentrate plus ground barley straw pellets can replace conventional feeding systems for light fattening lambs. Small Rum. Res..

[28] Owaimer A., Kraidees M., Al-saiady M., Zahran S., Abouheif M. (2004). Effect of Feeding Olive Cake in Complete Diet on Performance and Nutrient Utilization of Lambs. Asian-Australas J. Anim. Sci..

[29] Sharif M., Ashraf M.S., Mushtaq N., Nawaz H., Mustafa M.I., Ahmad F., Younas M., Javaid A. (2018). Influence of varying levels of dried citrus pulp on nutrient intake, growth performance and economic efficiency in lambs. J. Appl. Anim. Res..

[30] Awawdeh M.S., Dager H.K., Obeidat B.S. (2019). Effects of alternative feedstuffs on growth performance, carcass characteristics, and meat quality of growing Awassi lambs. Ital. J. Anim. Sci..

[31] Rodríguez R.B., de Frutos Fernández P., García F.J.G., Angulo G.H., Puente S.L. (2009). Effect of sodium bicarbonate supplementation on feed intake, digestibility, digesta kinetics, nitrogen balance and ruminal fermentation in young fattening lambs. Span. J. Agric. Res..

[32] Andrés S., Jaramillo E., Bodas R., Blanco C., Benavides J., Fernández P., González E.P., Frutos J., Belenguer A., Lopéz S. (2018). Grain grinding size of cereals in complete pelleted diets for growing lambs: Effects on ruminal microbiota and fermentation. Small Rum. Res..

[33] Benchaar C., Hassanat F., Gervais R., Chouinard P.Y., Julien C., Petit V., Massé D.I. (2013). Effects of increasing amounts of corn dried distillers grains with solubles in dairy cow diets on methane production, ruminal fermentation, digestion, N balance, and milk production. J. Dairy Sci..

[34] Firkins J.L., Yu Z., Morrison M. (2007). Ruminal nitrogen metabolism: Perspectives for integration of microbiology and nutrition for dairy cows. J. Dairy Sci..

[35] Carrasco C., Carro M.D., Fuentaja A., Medel P. (2017). Performance, carcass and ruminal fermentation characteristics of heifers fed concentrates differing in energy level and cereal type (corn vs. wheat). Span. J. Agric. Res..

[36] Bergen W.G., Henneman H.A., Magee W.T. (1973). Effect of dietary protein level and protein source on plasma and tissue free amino acids in growing sheep. J. Nutr..

[37] Bergen W.G. (1979). Free amino acids in blood of ruminants-physiological and nutritional regulation. J. Anim. Sci..

[38] Calsamiglia A., Ferret A., Reynolds C.K., Kristensen N.B., Van Vuuren A.M. (2010). Strategies for optimizing nitrogen use by ruminants. Animal.

[39] Lestingi A., Toteda F., Vicenti A., de De Marzo D., Facciolongo A.M. (2015). The Use of Faba Bean and Sweet Lupin Seeds Alone or in Combination for Growing Lambs. 1. Effects on Growth Performance, Carcass Traits, and Blood Parameters. Pakistan J. Zool..

[40] Lobón S., Joy M., Casasús I., Rufino-Moya P.J., Blanco M. (2020). Field Pea can be Included in Fattening Concentrate without Deleterious Effects on the Digestibility and Performance of Lambs. Animals.

[41] Xu Y., Li Z., Moraes L.E., Shen J., Yu Z., Zhu W. (2019). Effects of Incremental Urea Supplementation on Rumen Fermentation, Nutrient Digestion, Plasma Metabolites, and Growth Performance in Fattening Lambs. Animals..

